# Multiple Sclerosis between Genetics and Infections: Human Endogenous Retroviruses in Monocytes and Macrophages

**DOI:** 10.3389/fimmu.2015.00647

**Published:** 2015-12-24

**Authors:** Elena Morandi, Rachael E. Tarlinton, Bruno Gran

**Affiliations:** ^1^Clinical Neurology Research Group, Division of Clinical Neuroscience, School of Medicine, University of Nottingham, Nottingham, UK; ^2^School of Veterinary Medicine and Science, University of Nottingham, Nottingham, UK

**Keywords:** multiple sclerosis, human endogenous retrovirus, monocytes, macrophages, microglia, MS-associated retrovirus, Epstein–Barr virus, environmental factors

## Abstract

The etiology of multiple sclerosis (MS) is still unknown, but there is strong evidence that genetic predisposition associated with environmental factors can trigger the disease. An estimated 30 million years ago, exogenous retroviruses are thought to have integrated themselves into human germ line cells, becoming part of human DNA and being transmitted over generations. Usually such human endogenous retroviruses (HERVs) are silenced or expressed at low levels, but in some pathological conditions, such as MS, their expression is higher than that in the healthy population. Three HERV families have been associated with MS: HERV-H, HERV-K, and HERV-W. The envelope protein of MS-associated retrovirus (MSRV) from the HERV-W family currently has the strongest evidence as a potential trigger for MS. In addition to expression in peripheral immune cells, MSRV is expressed in monocytes and microglia in central nervous system lesions of people with MS and, through the activation of toll-like receptor 4, it has been shown to drive the production of proinflammatory cytokines, reduction of myelin protein expression, and death of oligodendrocyte precursors. In conclusion, the association between HERVs and MS is well documented and a pathological role for MSRV in MS is plausible. Further studies are required to determine whether the presence of these HERVs is a cause or an effect of immune dysregulation in MS.

## Introduction

Multiple sclerosis (MS) is one of the most common causes of neurological disability in young adults between the ages of 20 and 40 years. It is a chronic demyelinating disease of the central nervous system (CNS) characterized by inflammatory and degenerative changes in the brain and spinal cord. Pathological examinations of brains of patients with MS show characteristic perivascular inflammatory infiltrates, in particular T cells and macrophages, together with myelin breakdown and degeneration of axons (1). The etiology of MS is still unknown, but there is strong evidence that genetic predisposition associated with environmental factors can trigger the disease. Human endogenous retroviruses (HERVs) are integrated in the human genome and have been transmitted over generations. Usually HERVs are silenced or expressed at low levels, but in some pathological conditions, such as MS, their expression is higher than that in the healthy population.

In this review, we examine the expression of HERVs in monocytes/macrophages and their possible role in the pathogenesis of MS.

## Monocytes and Macrophages in MS

In the bone marrow, myeloid progenitor cells generate common monocyte–dendritic cell progenitors, which, in turn, yield blood monocytes and dendritic cell progenitors. Monocytes are blood mononuclear cells and are renewed continually from bone marrow hematopoietic stem cells throughout their life. Monocytes have the ability to mobilize and traffic to where they are needed through chemokines and leukocyte adhesion and trafficking molecules. The recognition of different chemokines is central for their function (2). Indeed during inflammation, monocytes migrate from the bloodstream into affected tissues, including the CNS, where they differentiate into “infiltrating” macrophages that are maintained either by local self-renewal signals or by interaction with the adaptive and innate immune system, such as toll-like receptors (TLRs) (3). Based on different environmental signals, macrophages can direct their phenotype into a number of functional phenotypes, including M1, proinflammatory macrophages (induced *in vitro* from monocytes by IFN-γ) and M2, quiescent/anti-inflammatory macrophages (induced by IL-4 or IL-13) (4). M1 have been involved in antigen presentation, cytotoxicity, also tissue remodeling, tumor destruction, and potentially tissue damage in neurodegeneration. By contrast, M2 participate in immune regulation, phagocytosis, cell survival signals, and tumor promotion (4). In reality, M1 and M2 are only two of many “poles” of macrophage differentiation, which encompasses a much broader transcriptional repertoire, encompassing at least nine distinct programs. These are influenced by a wide array of environmental signals (5).

Microglia have often been referred to as resident macrophages in the CNS; and only in the past few years, it has been possible to differentiate between monocyte-derived macrophages (MDM) and microglia. True microglia are resident mononuclear phagocytes of the brain parenchyma that originate during embryogenesis from the yolk sac and are maintained independently of hematopoietic stem cells. When the CNS is inflamed, microglia can differentiate into macrophages, whose functions are distinct from those of infiltrating monocytes. In the healthy brain, microglia physically contribute to brain development and homeostasis, including regulation of cell death, synapse pruning and elimination, neurogenesis, and neuronal surveillance (3).

In MS pathogenesis, which is helpfully modeled, with limitations, by the animal model experimental autoimmune encephalomyelitis (EAE), the destruction of myelin and axons as well as oligodendrocyte cell death are directly related to inflammation and the presence of monocytes/macrophages (6). Even though EAE is thought to be induced by T cells in most rodent and non-human primate models, the majority of CNS-infiltrating immune cells in such models are of myeloid origin. In the mouse, two main types of monocytes exist, including the infiltrating inflammatory monocytes (Ly6ChiCCR2 + CX3CR1lo) and the CNS resident monocytes (Ly6CloCCR2 − CX3CR1hi) (7). In EAE, only infiltrating CCR2+ Ly6Chi monocytes are rapidly recruited to the inflamed CNS prior to the disease onset and play a crucial role in the effector phase of the disease (8). Moreover, MHC class II-expressing macrophages are involved in the reactivation of pathogenic T cells in the subarachnoid spaces of the meninges and in the perivascular spaces of the blood–brain barrier (7). Again in EAE, the inhibition of macrophage inflammatory protein (MIP)-1α (CCL3), a ligand for CCR1 and CCR5, prevents the infiltration of macrophages into the CNS and the development of both acute and relapsing symptoms (9). Similarly in humans, active MS lesions are characterized by CCR1+/CCR5+ monocytes that are found in perivascular cell cuffs and in demyelinating lesions. Mononuclear phagocytes in early demyelinating stages comprise CCR1+/CCR5+ monocytes and CCR1−/CCR5− resident microglial cells that in later stages differentiate uniformly into CCR1−/CCR5+ phagocytic macrophages (10). Although macrophages in active MS lesions predominantly display M1 characteristics, a major subset of these cells also co-express M2 markers (11). Indeed, current pieces of evidence suggest that phenotypically similar macrophages in the CNS not only can contribute to the generation of inflammatory lesions and perform a pathogenic role in the demyelination process, but also can contribute to regenerative repair mechanisms to resolve inflammation (7).

Monocytes/macrophages have been implicated in inducing neural pathology in MS by secretion of toxic molecules, antigen presentation to cytotoxic T lymphocytes, and degradation of synapses (6). An additional proposed mechanism is by the expression of HERVs, as will be discussed in this review.

## Human Endogenous Retroviruses in MS

Retroviruses are enveloped viruses with single-strand positive RNA genomes. After the infection of the target cell, they reverse transcribe their RNA and integrate the resulting DNA product into the cellular chromosomes, forming a provirus. Occasionally, some types of retroviruses can infect germ line cells and colonize the host’s germ line by forming endogenous retroviruses. From 70 to 30 million years ago, various groups of retroviruses integrated into human germ line cells, becoming part of the human DNA and being transmitted through a Mendelian pattern over generations. Indeed up to 8% of the human genome is constituted of groups of HERVs ranging in copy number from one to many thousands. They are part of our history and evolution and although usually they retain only a passive role in the genome, in some cases, they have been associated with diseases (12).

**Figure 1:**
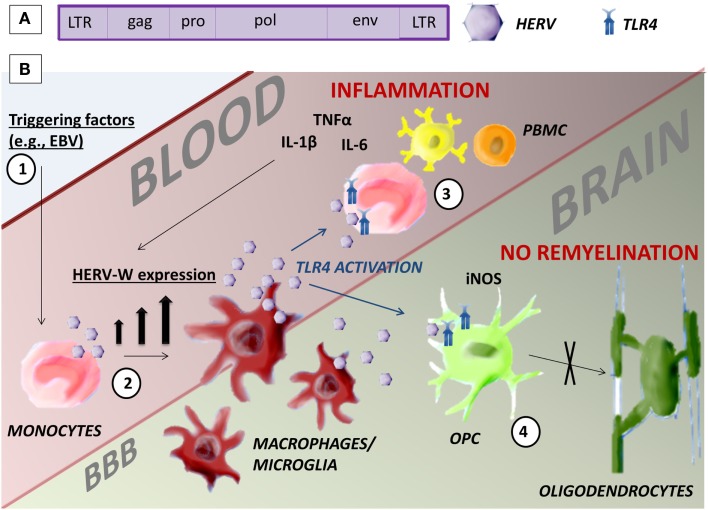
**Human endogenous retrovirus**. **(A)** HERV gene structure. **(B)** Possible mechanism of action of HERV-W/MSRV in monocytes that could be pathogenic in MS. (1) Environmental triggering factors, such as EBV infection, induce the expression of HERV-W in monocytes. (2) The differentiation of monocytes in macrophages and microglia increases the presence of HERV-W. (3) HERV-W activates TLR4-induced inflammation in the blood and further increases HERV-W expression. (4) TLR4 inhibits the maturation of OPC in oligodendrocytes in the brain with consequent lack of remyelination.

There is no universally recognized standard nomenclature for HERVs. They are classified into three categories on the basis of their sequence similarity with known infectious retroviruses: class I, II, and III, respectively, similar to gamma-retroviruses, beta-retroviruses, or spumaviruses. These classes are divided into several families in which the letter added to HERV (HERV-W, HERV-K, HERV-H, etc.) corresponds to the tRNA specificity of the primer binding site in the viral long terminal repeat (LTR). To date, according to this classification, 31 HERV families have been identified (12). HERVs have the same gene structure as exogenous retroviruses. Two LTR regions bound the genome that encodes the four major viral proteins: gag, the matrix and retroviral core; pol, the reverse transcriptase and integrase; pro, the protease; and env, the envelope (Figure 1A). HERV expression is regulated by the promoter and enhancer regions in the LTR.

Probably HERVs had initially the capacity of retrotransposition or reinfection, but over time, in the passage from one generation to the next, endogenous retroviruses have accumulated mutations or recombination events inactivating them and evolution may have selected only integrations that are harmless to humans (12). Although infectious ERVs have been identified in some mammals, such as cats, mice, and koalas, no HERVs have been shown to date to produce infectious virions. Some ERVs are indeed capable of initiating an infection into human cells, but they cannot complete their replication cycle due to mutations in their pol and gag genes (13). Even though mutations and control mechanisms that prevent their expression exist within human cells, such as epigenetic silencing and retrovirus restriction factors (APOBEC, Trim, and others), some HERVs maintain the ability to reverse transcribe, express proteins, and produce non-infectious viral particles. Between 7 and 30% of all HERV sequences in the genome may be transcriptionally active in normal tissues and cultured cells. However, the extent of expression varies from tissue to tissue and also between individuals. Nevertheless, their activity seems to be modulated in pathological conditions, such as cancer and autoimmune diseases (14).

Three main HERV families have been associated with MS. The first endogenous retrovirus found in MS samples in the 1980s was MS-associated retrovirus (MSRV) belonging to the HERV-W family (15). Most studies have since focused on this retroviral family. The majority reports an association between MSRV and MS. MSRVenv and MSRVpol RNA and protein have been found expressed at higher levels in serum (16–21), PBMC (21–26), and CSF (19, 27) of MS patients compared to healthy controls (HC) and other neurological disease (OND) patients. Some articles have also reported the presence of this retrovirus in the brain of MS patients, in particular within lesions (21, 24, 28), and the expression of these retroviruses correlated with disease progression (29, 30). On the other hand, a smaller number of studies have not reproduced the same association, finding similar expression levels of HERV-W in MS patients and control groups (31–33). This could be due to the use of different techniques or to genetic variation between different populations.

HERV-H (in particular the SNP rs391745 on HERV-Fc1) (34), HERV-K (in particular the polymorphism HERV-K18) (35), and HERES (Human T cell leukemia virus-related endogenous sequence) haplotype (36) have also been associated with MS in different studies.

The literature suggests a strong association between MS and HERVs, but are HERVs really involved in disease pathogenesis?

## Involvement of HERVs in MS Pathogenesis – The Importance of Monocytes/Macrophages

HERV-W has been studied by immunohistochemistry in healthy and MS human brains and the proteins pol, gag, and env were found to be upregulated, in particular in activated macrophages/microglia, astrocytes, and endothelial cells (21, 24, 28, 37). At a time when no clear discrimination between macrophages and microglia was possible, physiological expression of HERV-W/MSRVenv protein was detected in macrophages/microglia of both CNS gray and white matter and in certain blood vessel endothelial cells, whereas expression of HERV-W Gag antigens was observed in neurons (28). In particular, env antigen was found in macrophages in areas of recent demyelinating activity, but not in inactive lesions (28). According to a more recent study, these cells have a phenotype consistent with perivascular macrophages that express HLA-DR (21).

In peripheral mononuclear cells, HERV-W/MSRV RNA and proteins have been found expressed in monocytes/macrophages, NK, and B cells, and its expression is higher in MS patients; while in T cells, the presence of the retrovirus is undetectable at the RNA and protein level in both HC and MS (38). Similarly, HERV-H has been detected in B cells and monocytes, but not in T cells, with higher levels expressed in patients with active MS than stable MS and control groups (25).

Activation of primary human macrophages or differentiation of U937 monocytoid cells to induce a macrophage phenotype *in vitro* increases HERV expression in these cells (31, 38, 39). Monocyte differentiation leads to increased HERV-W, -H, and -K intracellular RNA expression, cell-free HERV sequences, and HERV RT activity in culture supernatants. Concurrent with elevated TNFα expression, altered expression of several HERV families has been observed in post-mortem brain tissue from patients diagnosed with MS (39).

Monocytes isolated from the peripheral blood of patients with active MS and stimulated *in vitro* with either PMA or LPS produced significant levels of gliotoxic activity, which correlated with increased HERV RNA and retrotranscriptase activity. This suggested a role for HERV expression in mediating gliotoxicity in MS monocytes/macrophages (40). The corresponding culture supernatants from these cells induced both astrocyte and oligodendrocyte cell death in primary mouse cortical cultures (40). Supernatant-induced cell death was also observed in immortalized astrocytes and oligodendrocytes and was concentration dependent (40).

Subsequently, using a vector expressing HERV-Wenv, one research group was able to infect human fetal astrocytes and monocytes/induced macrophages, but not oligodendrocytes, with HERV-W pseudoviruses. After infection, this group found production of the proinflammatory cytokine IL-1β from both astrocytes and macrophages and production of iNOS only from astrocytes (37). Conditioned medium from infected astrocytes induced cytotoxicity and death in human oligodendrocytes, but not neurons. Interestingly, antioxidants prevented this injury (37). A further study in human astrocytes showed binding of HERV-Wenv to the neutral amino acid transporter type 1 (ASCT-1). This interaction, in turn, induced the expression of an endoplasmic reticulum stress sensor and that of iNOS (41).

Another research group also found secretion of high amounts of TNFα, IL-1β, and IL-6 by monocytes from HC treated for 24 h with HERV-W/MSRVenv (42). Interestingly, the secretion of TNFα was blocked by anti-TLR4 and anti-CD14 ab, but not by anti-TLR2, suggesting that TLR4 could be involved in the proinflammatory effects of HERV-Wenv (42). Consistent with these observations, in chronic active MS brain lesions HERV-W/MSRVenv was detected in microglia/macrophages in proximity to TLR4-positive oligodendroglial precursor cells (OPC). The recombinant HERV-Wenv induced the production of iNOS and proinflammatory cytokines, such as TNFα, IL-1β, and IL-6 in cultured rat OPC, with associated reduction in myelin protein production and differentiation capacity (43). Moreover, HERV-W/MSRVenv was able to induce phenotypic and functional maturation of DC and confer them the potential to support the development of Th1-like effector lymphocytes (42). This pathogenic role of MSRV has also been demonstrated in MOG_35–55_-induced EAE in C57-BL/6 mice, where MSRVenv could substitute for mycobacterial lysate as a component of complete Freund’s adjuvant (CFA), and thereby inducing full-blown CNS disease (44). In this model, MSRVenv could also activate cells of the innate immune system, leading to a proinflammatory cytokine production through pattern recognition receptors TLR4 in association with CD14 (44).

The expression of HERV-W in monocytes could be a cause or a consequence of their activation and differentiation to macrophages. The trigger factor for HERV reactivation is still not clear, but there is evidence to suggest that other viral infections can act as co-factors. In culture, the expression of MSRV/HERV-W genes/proteins is activated by some viruses, such as Epstein–Barr virus (EBV), herpes simplex virus type 1, or by influenza virus (45). Interestingly, EBV is epidemiologically strongly associated with MS (46). This virus infects B cells and epithelial cells in over 90% of adult individuals. Primary infection usually occurs through contact with infected saliva and is usually asymptomatic during the acute phase in children; whereas in adolescents and adults, it is often symptomatic and can be present as infectious mononucleosis (IM). IM is usually a self-limiting disease resulting from a marked increase in circulating EBV-specific cytotoxic T lymphocytes and release of inflammatory cytokines. Interestingly, MS risk is two-to threefold higher among individuals with a history of IM (47). One recent study showed that binding of the EBV surface glycoprotein gp350 can activate the expression of MSRV/HERV-W in cells from blood and brain (U-87MG astrocytes) (38). In PBMC exposed to EBV gp350, HERV-Wenv is also expressed at higher levels in B cells and particularly in the monocyte/macrophage (M/M) cell compartment. The latter cells, particularly after differentiation to macrophages, are the most responsive to EBVgp350, expressing higher levels of HERV-Wenv than B cells (38). In another study, this group measured the expression of HERV-Wenv in EBV-uninfected healthy individuals, patients with IM, and individuals with high anti-EBNA-1 IgG titers, suggesting a past infection (48). Compared with uninfected individuals, IM patients had twofold higher frequency of HERV-W-positive B cells, and fourfold and 5.5-fold increase in NK cells and monocytes, respectively. In patients with past EBV infection, B cells showed similar percentages of HERV-W-positive B cells as those with IM (twofold higher than seronegative, uninfected individuals), intermediate percentages HERV-W-positive monocytes (2.6-fold higher than uninfected individuals), whereas NK cells were mostly HERV-W-negative (48).

## Conclusion

The etiology of MS is still unclear. Here, we briefly reviewed evidence supporting the hypothesis that HERVs could be the pathogenic factor linking an individual’s genetic makeup (and, therefore, their susceptibility to CNS autoimmunity) and infections as triggers of disease. Indeed, although HERVs are retroviruses, they are transmitted through generations and their integration loci differ between individuals, creating genetic variability. During the course of MS, there is high HERV-W/MSRV expression that increases at the time of relapses and through disease progression, confirming an association between HERV-W/MSRV and MS. In the peripheral blood, these retroviruses are mainly expressed by B cells and monocytes, particularly after differentiation of monocytes into macrophages. In MS lesions as well, HERV-W/MSRV has been found in macrophages/microglia. The expression of this retrovirus can lead to the production of inflammatory cytokines, cytotoxicity, and oligodendrocyte cell death through the activation of TLR4 (Figure 1B). Available evidence also indicates that HERV-W/MSRV expression is increased in cells infected by exogenous viruses, such as EBV, potentially providing a missing link between environmental triggers, and the immunopathogenic cascades leading to the MS lesions and to disease progression.

## Author Contributions

EM wrote the paper draft; RM and BG revised the paper. All authors read and approved the final manuscript.

## Conflict of Interest Statement

The authors declare that the research was conducted in the absence of any commercial or financial relationships that could be construed as a potential conflict of interest.
